# Xeroderma Pigmentosum Monozygotic Twins: Same Mutation Divergent Clinical Outcomes

**DOI:** 10.1155/humu/7247751

**Published:** 2026-10-07

**Authors:** José Pereira dos Santos Júnior, Maria Carolina Fernandes Cabral dos Santos, Lidiane Gomes da Silva, Wilson José da Silva Júnior, Camila Corradi, Veridiana Munford, Valdir de Queiroz Balbino, Túlio de Lima Campos, Carlos Frederico Martins Menck, Jaqueline de Azevêdo Silva

**Affiliations:** ^1^ Laboratory of Genetic and Human Molecular Biology Department of Genetic Federal University of Pernambuco Recife Pernambuco Brazil ufpe.br; ^2^ Keizo Asami Institute Federal University of Pernambuco Recife Pernambuco Brazil ufpe.br; ^3^ DNA Repair Laboratory Institute of Biomedical Sciences Department of Microbiology University of São Paulo São Paulo Brazil usp.br; ^4^ Laboratory of Bioinformatics and Evolutionary Biology Department of Genetic Federal University of Pernambuco Recife Pernambuco Brazil ufpe.br; ^5^ Bioinformatics Core Facility Aggeu Magalhães Institute Oswaldo Cruz Foundation Recife Pernambuco Brazil fiocruz.br

**Keywords:** genomic discordance, monozygotic twins, mutational signatures, whole-genome sequencing, xeroderma pigmentosum

## Abstract

Xeroderma pigmentosum (XP) is a rare monogenic disorder characterized by UV sensitivity and skin cancer predisposition, caused by mutations in the NER pathway or the gene encoding DNA polymerase eta (Pol *η*), which acts in translesion synthesis (TLS). Although XP is a monogenic disorder, differences in clinical severity may occur even between monozygotic twins. Here, we present monozygotic twins (M3 and M6) with the XP variant (XP‐V) displaying differences in clinical severity despite sharing similar environments and habits. Using whole genome sequencing (WGS), we evaluated their mutational burden and the impact of therapeutic strategies. Our results confirmed their kinship and the expected presence of the same *POLH* mutation in both patients. The somatic mutation profiles showed that Patient M3 harbored a significantly higher number of somatic mutations (*p* ≤ 0.0001), including the exclusive detection of the ID8 signature. Despite these differences, the mutational spectrum remained remarkably consistent between twins (cosine similarity > 0.98). In addition, as one of the patients was submitted to cisplatin (CDDP) therapy, we confirmed in vitro the impact of CDDP on the cell cycle of TLS‐deficient cells. Therefore, the analyses of the genome sequences of these twin XP‐V patients revealed that during their lifetime endogenous DNA damage and therapeutic treatments may lead to high levels of mutations and phenotype divergency in their genome.

## 1. Introduction

Xeroderma pigmentosum (XP) is a rare autosomal recessive disease characterized by ineffective DNA repair, affecting approximately 1 in 250,000 (United States) to 1 in 450,000 (Western Europe) live births. XP is caused by gene mutations leading to impairment in the nucleotide excision repair (NER) pathway—responsible for the removal of bulky DNA lesions—or the translesion synthesis (TLS) pathway. The TLS pathway acts as an alternative mechanism to bypass unrepaired DNA damage that stalls the replication fork. This process involves specific polymerases, such as DNA polymerase *η* (Pol *η*), whereas mutations in the *POLH* gene result in the XP variant (XP‐V) phenotype [1].

Different gene mutations within these pathways, as well as cumulative sun exposure, result in distinct clinical features, including increased sensitivity to UV rays and skin hyperpigmentation in areas exposed to the sun. Additionally, it increases the risk of developing tumors, with individuals under the age of 20 being 2000‐fold more likely to develop melanomas and, in general, they face higher than 10,000‐fold increased risk to develop basal cell and squamous cell carcinomas [2–5].

The diagnosis of XP is primarily based on clinical examination and is rarely confirmed by specific genetic tests in Brazil. Currently, whole exome sequencing (WES) is the most specific genetic test available. However, it requires specialized professionals for genome assembly and high‐capacity data processing infrastructure, both of which are financially prohibitive for most patients in Brazil. Nevertheless, the lack of WES analysis may limit treatment options, as identifying the specific altered gene is essential for developing mutation‐targeted therapeutic strategies [6, 7].

Preventively, patients must use high‐SPF (sun protection factor) sunscreen, wear sunglasses, long‐sleeved clothing, and hats. Darker colored fabrics are also recommended to better block UV radiation. When possible, patients should adhere to nocturnal habits to minimize sun exposure [8]. Here, we describe a case of identical twins with XP syndrome who display markedly different clinical symptoms. Both patients carry a homozygous mutation in the *POLH* gene, leading to a diagnosis of XP‐V, but received different chemotherapy strategies to manage disease′s complication. After whole genome sequencing (WGS), we identified a highly significant difference of exclusive somatic single nucleotide variants (SNVs) in both patients. Patient M3, who experienced greater clinical severity and had received cisplatin (CDDP)–based chemotherapy, exhibited a higher number of exclusive somatic mutations.

## 2. Material and Methods

### 2.1. Samples

This study included the investigation of two patients—probands—evaluated and followed up at the Children′s Cancer Institute of Agreste (Caruaru, Pernambuco, Brazil) (S1) with clinical diagnosis of XP. The proband′s parents were also included in the study. The samples were obtained after signed informed consent forms were provided by the patients and their parents.

A total of 4 mL peripheral blood from the parents M4 and M5 (self‐reported as consanguineous) and from monozygotic (MZ) twins M3 and M6 with XP clinical signs were collected in tubes containing EDTA. The collection process was accompanied by nursing and psychology professionals to minimize discomfort for those involved.

### 2.2. DNA Extraction and Genotyping

DNA extraction from whole blood was performed using the ReliaPrep Blood gDNA Miniprep System (Promega, Madison, United States). The DNA extracted from each sample was quantified in the NanoDrop 2000 spectrophotometer (Thermo Fisher Scientific, Waltham, Massachusetts, United States) and was subsequently subjected to agarose gel electrophoresis to assess DNA integrity.

The family was genotyped to confirm kinship, including the zygosity of the twins. Because the twins displayed differences in their clinical appearance, monozygosity could not be reliably inferred based on physical examination alone. The Multi‐user Genomics and Transcriptomics Platform of the Federal University of Pernambuco performed the test using the PowerPlex Fusion 6C STR (Promega, Madison, United States) and PowerPlex Y23 (Promega, Madison, United States) kits, following the manufacturer′s recommendations. These kits are specific to the following genetic markers: D3S1358, D1S1656, D2S441, D10S1248, D13S317, PENTA E, D16S539, D18S51, D2S1338, CSF1PO, PENTA D, TH01, vWA, D21S11, D7S820, D5S818, TPOX, D8S121S39, 39S39S, SE33, D22S1045, DYS391, FGA, DYS576, DYS570, and D6S1043. To perform the statistical analysis and interpretation of the DNA profiles, the BigASystem software was used to confirm the relationship of kinship and zygosity [9].

### 2.3. WGS

WGS was performed (DNBSEQ‐G400, MGI tech) to investigate mutagenic processes accumulated independently in each twin, from zygotic development to the time of sample collection, in addition to identifying the causative germline mutation responsible for the XP‐V phenotype. Parental samples were also sequenced to enable filtering of shared germline variants in each twin.

### 2.4. Somatic Variant Calling

Raw FASTQ sequences were quality‐checked with FastQC and aligned to the GRCh38 reference genome using BWA‐MEM. The variant call was performed following the steps described in the GATK Best Practices [10] for somatic short variant discovery (SNVs + indels), using Mutect2 (GATK v4.6.1.0) in tumor‐only mode and with the following optional arguments: –germline‐resource, –panel‐of‐normals. The germline file and the panel of normals were obtained from the GATK Resource Bundle (Broad Institute, gs://gatk‐best‐practices/somatic‐hg38/).

After the variant calling filtering was performed with FilterMutectCalls to distinguish true somatic variants from technical artifacts, we only retained the high‐confidence variants. To remove the background of germline mutations shared between twins and their parents, variants exclusive to each individual were retained. This strategy resulted in sets of somatic mutations unique to each twin, excluding variants detected in the co‐twin or in parental samples. Variants with a read depth of less than 10x (DP ≥ 10x) were excluded to ensure high‐quality calls.

To minimize false‐positive differences between the twins related to sequencing coverage, variants were strictly filtered based on genomic callability. Coverage profiles were generated for each sample using mosdepth (v.0.3.10) [11], defining callable regions as those with a depth of at least 10x and only variants located within the intersection for both M3 and M6 were retained. The high‐confidence intersection of identifiable regions between the two samples spanned 2700.05 Mb, representing approximately 91.9% of the non‐N bases of the GRCh38 reference genome, ensuring that the observed differences in mutational load and substitution profiles were not influenced by local disparities in sequencing depth.

After the filtering stage, annotation was performed using ANNOVAR (v. Oct. 2010) [12] with the avsnp150 and RefGene databases, both obtained from the tool′s official website.

### 2.5. Mutational Signatures

To characterize the mutational spectra of M3 and M6, the R/Bioconductor package SomaticSignatures (v2.44) [13] was employed. Using the *mutationContext* function, the genomic neighborhood of each single base substitution (SBS) was retrieved from the human reference genome hg38, and the *motifMatrix* function was used to construct a count matrix for the 96 trinucleotide contexts. Finally, the observed mutational spectra for both samples were obtained, where motifs exhibiting significant differential enrichment between the twins were explicitly labeled.

Subsequently, to identify the contribution of characteristic patterns of somatic mutations, the R/Bioconductor package MutationalPatterns (v3.14) was used [14], fitting the observed mutational profiles against known mutational signatures cataloged in the COSMIC database (Version 3.5) [15]. Signatures contributions were estimated using a readjustment method that mathematically optimizes the fit to the observed mutational profiles. To ensure a parsimonious model and minimize overfitting, a rigorous selection approach was implemented using the *fit_to_signatures_strict function* with a threshold of max_*d* *e* *l* *t* *a* = 0.04 *8.* This ensured that only signatures that provide a statistically significant improvement in model fit are retained in the final analysis. The relative contribution of each signature is shown in the respective plot.

### 2.6. Motif Analysis

To characterize the local genomic context surrounding SNVs and mutational motifs, we employed a log‐probability analysis using the pLogo platform (Version 1.2.0) [16] and standardized the variants to use the pyrimidine reference. The pLogo approach scales each nucleotide according to its statistical significance relative to a background distribution, with hg38 serving as the reference genome in this analysis. In the graph, the *y*‐axis represents the logarithm of the binomial probability, where nucleotides plotted above the horizontal midline indicate statistically significant enrichment (overrepresentation) and those below indicate depletion (underrepresentation). To ensure rigorous statistical inference, the significance threshold was set at ± 2.68, corresponding to a Bonferroni‐corrected *p* value of *p* < 0.05. The central position (0) was fixed according to the variant of interest, whereas the flanking positions (−3 to +3) were analyzed for nonrandom base preferences. This method allowed the identification of specific sequence signatures, such as the enrichment of certain bases in positions adjacent to the mutation site.

### 2.7. Cell Lines and Cell Culture Conditions

Two SV40‐transformed human fibroblast cell lines were cultured: XP‐V comp (control line), complemented with the XPV/POLH gene, and XP‐V (test line), deficient in TLS [17, 18]. The cells were maintained in Dulbecco′s Modified Eagle Medium (DMEM) High Glucose (LGC Biotechnologies, Cotia, São Paulo, Brazil) under a humidified atmosphere with 5% CO_2_ at 37°C, supplemented with 10% fetal bovine serum (FBS) (Life Technologies, Carlsbad, California, United States) and 1% antibiotic–antimycotic solution (0.1‐mg/mL penicillin, 0.1‐mg/mL streptomycin, and 0.25‐mg/mL fungizone; Life Technologies).

### 2.8. Cell Lines′ Authentication

The cell lines had their WES previously [19], confirming their common origin, the mutation in the POLH gene, and the complementation of this mutated allele in the complemented cells. Moreover, functional experiments, such as cell sensitivity to UVC radiation, S‐phase progression after DNA damage, and TLS, confirmed the expected phenotypes.

The human cell lines used in this study are part of a collection approved for use by the Ethical Committee for Research in Humans of the Institute of Biomedical Sciences, USP (Of. CEPSH 142.11).

### 2.9. Cell Treatment

The cells were treated with different concentrations of CDDP in micromolar (*μ*M) (1, 5, and 10) and tested with exposure times of 2 and 72 h. At the end of the exposure time to CDDP, the medium was removed, then cells were washed with 1x PBS and replaced with DMEM supplemented with 10% FBS and 1% antibiotic‐antimycotic solution.

### 2.10. Tetrazolium Metabolization Test (XTT Assay)

Approximately 50,000 cells per well from each cell line (XP‐V comp and XP‐V) were plated in 24‐well plates, with triplicates for each CDDP concentration to be tested, in addition to the negative control (0). After cell treatments, the XTT solution was prepared using 1x PBS, XTT salt, and electron coupling solution, according to the number of wells per plate, with three blank wells and one extra well.

The culture medium was removed and replaced with 200 *μ*L of the XTT solution. The cells were then incubated for 40 min in a humidified incubator with 5% CO2 at 37°C to metabolize the tetrazolium. Subsequently, the cells were transferred to a 96‐well plate for absorbance reading using the GloMax Discover Microplate Reader (Promega, Madison, United States). Finally, the absorbance ratio was used to calculate the percentage of cell viability.

### 2.11. Flow Cytometry

Approximately 300,000 cells per line were seeded in duplicate for both NC and CDDP (10 *μ*M) groups, with a treatment duration of 2 h. Following medium replacement, cells were collected and fixed at 24‐ and 72‐h posttreatment. Samples were stained with propidium iodide (PI) as previously described [17, 20] and analyzed using a BD Accuri C6 Plus Personal Flow Cytometer (BD Biosciences, Franklin Lakes, New Jersey, United States). Data acquisition and analysis were performed using the *flowCore* package in R software (Version 4.5.1) [21], applying default settings for PI‐based cell cycle analysis.

### 2.12. Statistical Analysis

Statistical analyses were performed using R software (Version 4.5.1). For XTT cell viability, a two‐way ANOVA was employed to determine the significance of differences between treatments. For flow cytometry assays, data were evaluated for biological consistency, and extreme outliers were identified using Grubbs′ test. One outlier in the XP − V comp + CDDP (24 h) group (7.87% vs. a group mean of 0.30%) was excluded to satisfy the assumption of homogeneity of variance (Levene′s test) required for ANOVA. The removal of this data point ensured the statistical robustness of the downstream comparisons. Post hoc analyses were conducted using Tukey’s multiple comparison test for XTT results and Sidak′s multiple comparison test for flow cytometry data.

Parallel to the cellular assays, mutational profiles from WGS data were compared between the twins. The mutational burden was evaluated using a chi‐square goodness of fit test, testing the null hypothesis of an equal distribution of variants between both twins. To investigate differences in the relative frequencies of substitution classes, Pearson′s chi square test was applied to 2 × 2 contingency tables for each mutation type. To account for multiple tests among the six substitution classes, *p* values were adjusted using the Benjamini–Hochberg false discovery rate (FDR) method and for all analysis, statistical significance was defined as follows: ns, not significant; *p* > 0.05;  ^∗^ *p* ≤ 0.05,  ^∗∗^ *p* ≤ 0.01,  ^∗∗∗^ *p* ≤ 0.001, and  ^∗∗∗∗^ *p* ≤ 0.0001.

## 3. Results

M3 and M6, male MZ twins (Figure 1A), aged 28, have resided in the rural area of Agrestina, located in the Agreste region of Pernambuco, Northeast Brazil (Figure S1), throughout their lives. They share the same household and engage in identical agricultural activities centered on small‐scale cattle farming and dairy management (raising and milking cows for milk production), with outdoor work routinely performed without adequate sun protection. A detailed review of their environmental and occupational history revealed no exposure to pesticides or other hazardous agricultural chemicals in either twin or their family members. Regarding lifestyle factors, both twins reported similar dietary habits, with no history of tobacco use in either individual or among their family members.

**Figure 1:**
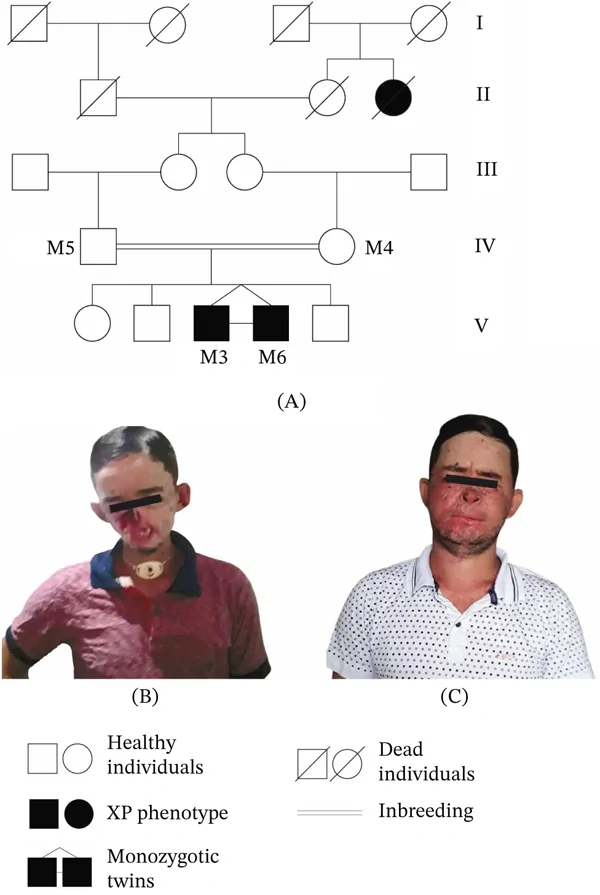
Genealogy and phenotype of *XPV* twins from Pernambuco (Brazil). *Description:* (A) Pedigree of the family showing the affected individuals and the reported occurrence of a great‐aunt with a clinical history suggestive of XP. (B) Clinical photograph of Patient M3 after surgical resection of an invasive squamous cell carcinoma involving the right maxilla and gingival region, resulting in partial facial tissue loss and reduced maxillary mobility. (C) Clinical photograph of Patient M6 following surgical removal of an invasive squamous cell carcinoma involving the left orbit, resulting in permanent loss of vision in the affected eye.

M3 (Figure 1B) exhibits the typical clinical manifestations of XP and experienced a more severe clinical course (Table 1). His management included four cycles of chemotherapy (CDDP, 5‐fluorouracil [5‐FU], and docetaxel [DCT]) and six sessions of radiotherapy for the treatment of a large facial tumor. Although formal nutritional assessments prior to diagnosis were unavailable, the clinical history revealed no relevant differences in dietary habits or nutritional status before disease onset. Following partial right‐sided facial resection and oncological therapy, he developed reduced maxillary mobility, which severely impaired oral intake and contributed to weight loss and compromised nutritional status. M6 (Figure 1C) also exhibited the typical clinical manifestations of XP but experienced a comparatively less severe clinical course (Table 1). He likewise underwent chemotherapy, although details of the treatment protocol were unavailable.

**Table 1:** Clinical manifestations observed in the two patients with XP‐V.

Clinical manifestation	Patient M3	Location	Patient M6	Location
Dry, rough, and atrophic skin	Present	All sun‐exposed skin extension	Present	All sun‐exposed skin extension
Solar lentigines and ephelides	Present	Head and neck	Present	Head, neck, and upper limbs
History of sunburns	Present	Exposed areas	Present	Exposed areas
Neurological damage	Absent	—	Absent	—
Squamous cell carcinoma	Present	Face	Present	Face and metastatic
Basal cell carcinoma	Present	Face	Present	Neck
Superficially invasive squamous cell carcinoma	Present	Right eye	Absent	—
Nodular melanoma	Present	Right eye	Absent	—
Squamous cell papilloma	Present	Left eyelids	Absent	—
Basosquamous carcinoma	Absent	—	Present	Nose and lips
Invasive squamous cell carcinoma	Absent	—	Present	Left eye socket
Lymph nodes	Present	Metastatic	Present	Metastatic

*Note:* The table summarizes the clinical manifestations identified in Patients M3 and M6 and their corresponding anatomical locations during clinical follow‐up.

Genotyping of the probands and their parents confirmed the parental relationship between the research participants with a probability > 99.9*%*, according to Hummel′s classification. The zygosity test confirmed the probability of patients being MZ with a probability exceeding 99.99%.

We identified by WGS a thymine insertion in homozygosity at *POLH* exon 6 in both patients. This insertion, *POLH* c.672_673insT, results in a frameshift mutation culminating in a premature termination codon (p.Leu225Serfs ^∗^33). This variant (rs772778835) is deleterious to gene function and was previously described in a specific community with a genetic cluster of XP‐V patients in the northeast region of Brazil [22]. This variant has populational low frequency (0.01624) in heterozygosity according to the Genome Aggregation Database (gnomAD) (https://gnomad.broadinstitute.org/).

Based on a target size of 2700.05 Mb, we identified 18,574 exclusive somatic mutations in Patient M3, of which 11,132 were SNVs. Additionally, this patient carries 7442 indels, comprising 3403 insertions and 4039 deletions. In Patient M6, the exclusive somatic mutational burden was 15,392, consisting of 7781 SNVs and approximately 7611 indels, of which 4070 were insertions and 3541 were deletions. The relative distribution of mutations for each twin is represented in Figure 2.

**Figure 2:**
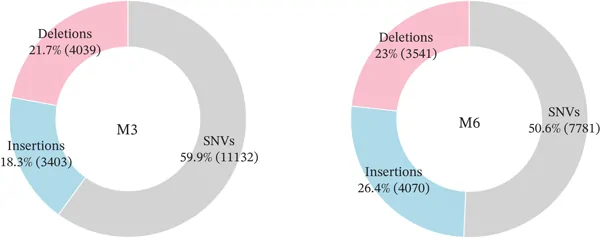
Comparative analysis of mutational types*. Note:* The graphics show the proportion (%) and absolute number (*n*) of single nucleotide variants (SNVs), insertions, and deletions detected in each patient.

A highly significant difference in the total number of substitutions was observed between the two patients (*p* ≤ 0.0001) (Figure 3). However, when mutations were analyzed by type, their distribution was similar in both twins, and no statistically significant differences were detected.

**Figure 3:**
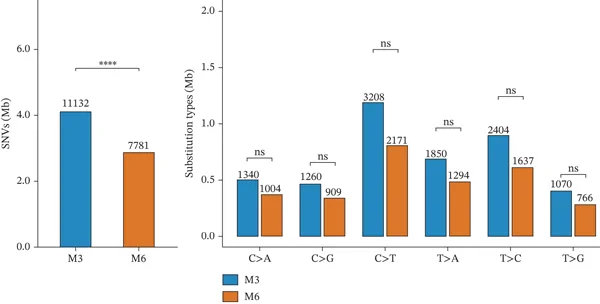
Substitution profiles in Patients M3 and M6*. Note:* Number of single nucleotide variants (SNVs) per megabase (Mb) and the distribution of substitution classes (C > A, C > G, C > T, T > A, T > C, and T > G) per megabase in Patients M3 and M6. Data are presented as mutation counts per Mb. Statistical significance is indicated as  ^∗∗∗∗^ *p* ≤ 0.0001; ns, not significant.

Following filtering for unique mutations, the total number of variants identified in M3 was 11,132, distributed as follows: 8,521 intergenic, 1,550 intronic, 22 exonic, 77 in the 3 ^′^ UTR, 8 in the 5 ^′^ UTR, 58 upstream, and 54 downstream. Additionally, 66 were categorized as ncRNA exonic and 776 as ncRNA intronic. In contrast, M6 carried 7781 variants, comprising 6031 intergenic, 1060 intronic, 33 exonic, 47 in the 3 ^′^ UTR, 6 in the 5 ^′^ UTR, 52 upstream, and 42 downstream, along with 40 ncRNA exonic and 470 ncRNA intronic variants. The report on total variants of twins is available in Tables S1 and S2.

To further characterize the mutational profile, we calculated the transition to transversion (Ti/Tv) ratio, indicating 1.02 for M3 and 0.96 for M6. The mutational spectra of both patients were predominantly characterized by C > T transitions, with M3 and M6 displaying 3208 and 2171 absolute variants, respectively. Despite these numerical differences, the relative contribution of C > T substitutions followed a consistent pattern between the twins. Within CpG sites, these transitions accounted for 34.16% in M3 and 31% in M6. A similar distribution was observed in YpY regions, where M6 showed a slightly higher contribution (56.06%) compared with M3 (52.43%). For all other contexts, the distribution remained stable, averaging 13.4% for M3 and 12.94% for M6 (Table S3).

To evaluate the consistency of the mutational process, we compared the mutational spectra across the 96 SBS contexts between M3 and M6. The analysis revealed a high cosine similarity score (*r* > 0.98), demonstrating a strong concordance in the mutational signatures observed in both twins (Figure 4). This result underscores that the mutational process is highly conserved across the patients, despite variations in their total mutational burden.

**Figure 4:**
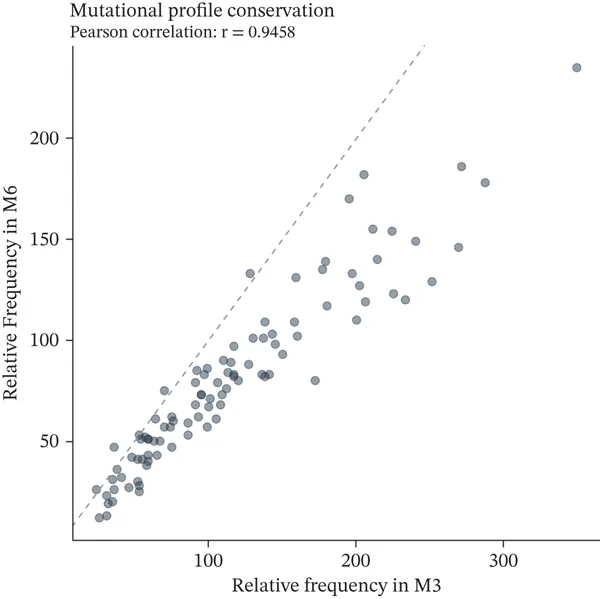
Cosine similarity analysis of mutational profiles between M3 and M6. *Note:* Relative frequencies of substitution types in Patient M3 (*x*‐axis) and Patient M6 (*y*‐axis). Cosine similarity was used to quantify the similarity between the two mutational profiles. The dashed line represents the line of identity (*y* = *x*), and the cosine similarity score (0.9862) is shown in the figure.

Although variants were distributed across the entire spectrum, some trinucleotide context exhibited a relative contribution exceeding 0.02 for each sample. Notably, the ACA, ACG, and GCG contexts for C > T were prevalent in both twins, whereas the CCG and TCG triplets (also for C > T) were distinctively prominent only in M3, whereas CCT and TCT were found only in M6. Additionally, in the T > A spectrum of M3, the TTA context stood out, whereas for T > C, the ATA, ATG, and TTT triplets were the most significant (Figure 5). Other triplets are described in Table S4.

**Figure 5:**
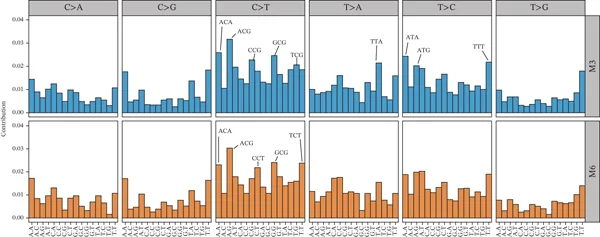
Somatic mutational spectra in M3 and M6*. Note:* Relative contribution of the 96 trinucleotide substitution contexts identified in Patients M3 and M6. Mutation classes are grouped according to the six base substitution types (C > A, C > G, C > T, T > A, T > C, and T > G), with bars representing the relative frequency of each trinucleotide context.

Since the mutational spectra were compared between the XP‐V twins, M3 and M6 were analyzed relative to the hg38 reference genome using pLogo to identify position‐specific enrichment. For substitutions from cytosine, we observed similar motifs for the occurrence of C > A, being AAAC‐AT for M3 and AA‐CAA for M6. Both are rich in adenosine. Similar motifs were also observed for the occurrence of C > T, being WTACGTT (where W denotes A or T, according to IUPAC nomenclature) for M3 and ATACG for M6. Both have high G enrichment at the +1 position (Figure 6).

**Figure 6:**
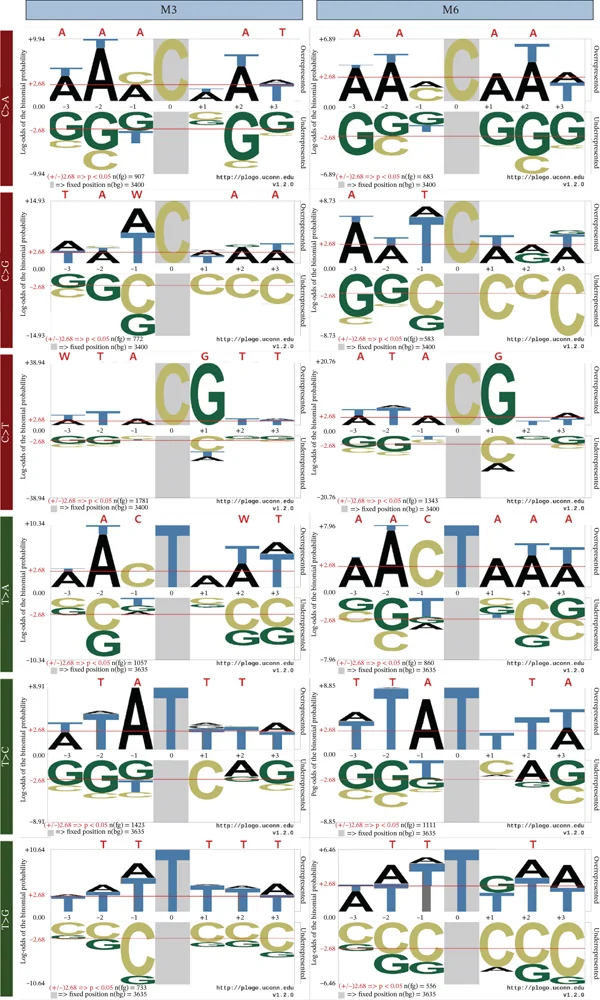
Probability logo (pLogo) of the sequential context for mutations in M3 and M6. *Note:* Visualization of the expanded genomic context for variants found in M3 and M6 in relation to the hg38 reference genome. The horizontal line positioned at 2.68 corresponds to the threshold for statistical significance based on the Bonferroni correction; bases at significantly enriched positions (*y* > 2.68; *p* < 0.05) are highlighted above the context. According to IUPAC nomenclature, W represents A or T. Position 0 (gray) denotes the fixed reference base: cytosine for C > A, C > T, and C > G substitution classes (dark red label), and thymine for T > A, T > C, and T > G substitution classes (dark green label).

For substitutions from thymine, we observed a high occurrence of substitutions in T‐rich regions. Considering the T > C variants, we observed the TATTT motif for M3 and TTAT‐TA for M6, and for T > G, the TTTTTT motif for M3 and TTT‐T for M6 (Figure 6). This method allowed the identification of specific sequence signatures, such as the enrichment of certain bases in positions adjacent to the mutation site.

The relative and absolute contributions of COSMIC SBS signatures show a similar composition pattern across certain SBSs. The mutational landscape of both M3 and M6 is primarily characterized by major contributions from SBS3 and SBS25; however, these contributions are approximately 20% and 13% higher in M6, respectively (Figure 7). M3 also exhibits contributions from SBS6 and SBS37, diverging from M6, which shows no additional signature contributions. Notably, SBS3, SBS6, and SBS25 represent the most significant signatures identified in these patients.

**Figure 7:**
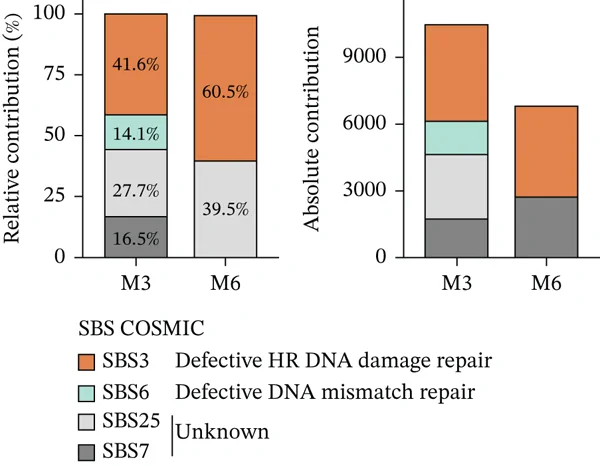
SBS COSMIC signatures contribution for each twin. *Note:* Relative contribution (%) and absolute contribution of the COSMIC SBS mutational signatures identified in Patients M3 and M6.

The indels profiles of the patients were evaluated; overall, the difference in the total number of indels was not statistically significant. However, when analyzed individually, a predominance of deletions was identified in M3, whereas M6 predominantly harbors insertions in its genome. These individual different patterns were highly significant (*p* ≤ 0.0001) as observed in Figure 8.

**Figure 8:**
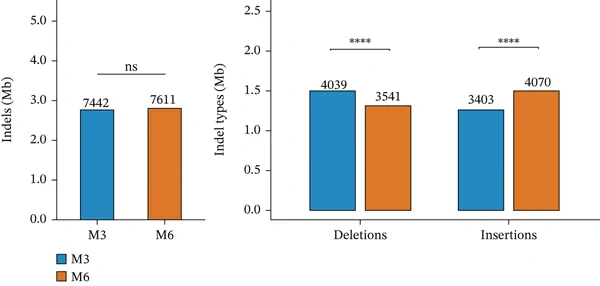
Insertions and deletions in Patients M3 and M6. *Note:* Absolute number of insertions and deletions identified in each patient. Statistical significance is indicated as  ^∗∗∗∗^ *p* ≤ 0.0001; ns, not significant.

When evaluating COSMIC ID signatures (Figure 9), only one similarity was found: Both M3 and M6 share ID2, although its contribution is higher in M6 at 30.2%. The remaining signatures diverge, with M3 showing contributions from ID8 and ID14, whereas M6 exhibits ID1 and ID12. Although ID1 and ID2 are associated with polymerase slippage at homopolymer tracts, ID8 is linked to DNA double‐strand breaks (DSBs) repaired by nonhomologous end joining (NHEJ). For ID classification, COSMIC only considers small indels, typically defined as indels between 1 and 50 base pairs in length. Therefore, the count of ID signatures differs from the total number of indels.

**Figure 9:**
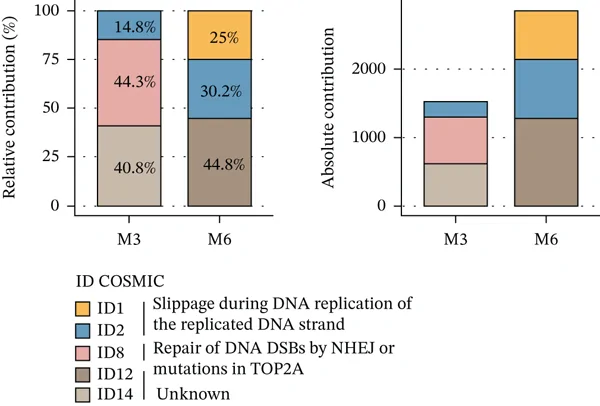
ID COSMIC signatures contribution for each twin. *Note:* Relative contribution (%) and absolute contribution of the COSMIC ID mutational signatures identified in Patients M3 and M6.

To assess the influence of CDDP in order to simulate the effects of this chemotherapeutic agent in the XP‐V patients, we used XP‐V cells for CDDP exposure. The results indicated that XP‐V cells are more sensitive than XP‐V complemented cells. During exposure to the CDDP for 2 h, the viability of XP‐V cells decreased considerably when compared with the negative control (0). When the exposure lasted for 72 h, the reduction in XP‐V cell viability was even more pronounced, with just over 4% survival observed at a concentration of 1 *μ*M (Figure 10A). A slight reduction in the viability of XP‐V comp cells was observed, but viability remained above 80% at all concentrations tested for 2 h. After 72 h of exposure, viability was also reduced considerably, but with a higher survival rate than XP‐V cells (Figure 10B). These results confirm the high sensitivity of XP‐V cells to CDDP, compared with cells proficient for Pol *η*.

**Figure 10:**
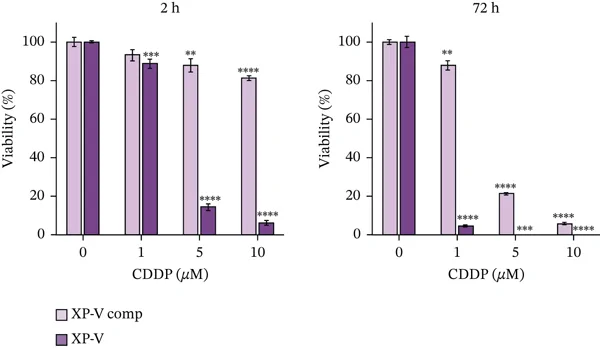
Cell viability of XP‐V and XP‐V comp cells after CDDP exposure. *Note:* Cell viability of XP‐V and XP‐V comp fibroblasts was evaluated using the XTT assay. Cells were exposed to CDDP for 2 h, and cell viability was measured 72 h after treatment. Cell viability is expressed as a percentage of untreated control cells. Data are presented as mean ± SD. Comparisons were performed between XP‐V and XP‐V comp cells at each CDDP concentration. Statistical significance is indicated as  ^∗∗^ *p* ≤ 0.01,  ^∗∗∗^ *p* ≤ 0.001 and  ^∗∗∗∗^ *p* ≤ 0.0001.

Cell cycle progression was not altered 24‐h post‐CDDP treatment (10 *μ*M for 2 h). At 72‐h posttreatment, XP‐V comp cells successfully resumed the cell cycle, as evidenced by the recovery of G1 and G2/M peaks. Conversely, XP‐V cells remained stalled at the G1/S transition, indicating a failure to bypass S‐phase (Figure 11).

**Figure 11:**
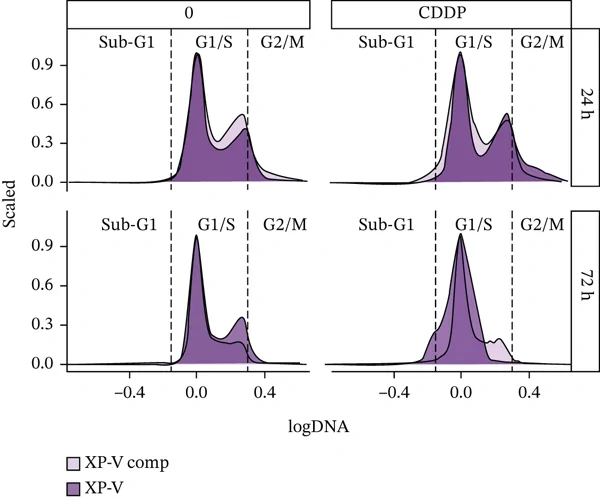
Cell cycle assessment 24 h and 72 h after exposure to CDDP*. Note:* Representative DNA content histograms obtained by flow cytometry after PI staining. Cells were exposed to 10 *μ*M CDDP for 2 h and analyzed at 24‐ and 72‐h posttreatment. Histograms show the distribution of cells in the sub‐G1, G1/S, and G2/M phases.

## 4. Discussion

Phenotypic variability among individuals carrying identical pathogenic variants has been reported across several NER disorders, including XP and Cockayne syndrome (CS). North African studies of XP have demonstrated that genotype–phenotype correlations are often less predictable than expected. For example, Tunisian patients carrying the same *ERCC5* pathogenic variant exhibited a considerably milder XP‐G phenotype than classically described, highlighting that genotype–phenotype correlations in XP are not always straightforward and that the causal mutation alone may not fully determine disease expression. Similar observations have been reported in CS, another NER disorder, in which Tunisian siblings carrying identical *ERCC8* mutations displayed marked differences in clinical severity despite sharing the same pathogenic variant [23, 24].

Our findings extend these observations by demonstrating differences in clinical severity between MZ twins carrying the same homozygous pathogenic *POLH* variant. Unlike studies involving siblings, MZ twins largely eliminate differences in genetic background, thereby highlighting the contribution of additional modifiers, including cumulative UV exposure, photoprotective behavior, epigenetic regulation, and somatic variation, to disease severity.

Similar variability in clinical severity has also been described in MZ twins affected by monogenic disorders [25]. Consistent with these observations, at the time of their clinical diagnosis of XP at 6 years, M3 presented actinic keratosis, whereas M6 had already developed a basal cell carcinoma. Over the years, M3′s condition worsened considerably, developing secondary complications due to the severity of the tumors and the chemotherapy treatment adopted, resulting in melanoma at age 20. Although M6 also developed the characteristic cutaneous manifestations of XP‐V, including metastatic disease, his overall clinical course was less severe, with a lower tumor burden and without the extensive surgical sequelae and early melanoma observed in M3.

XP patients exhibit a markedly increased susceptibility to UV‐induced skin cancers because defective DNA repair promotes the accumulation of somatic mutations. Recent genomic studies estimate that XP is associated with an approximately 10,000‐fold increased risk of nonmelanoma skin cancer and a 2000‐fold increased risk of melanoma compared with the general population [26]. Although melanoma is a well‐recognized complication of XP, published data specifically addressing nodular melanoma according to XP complementation group or *POLH* genotype remain scarce. Likewise, evidence regarding survival outcomes of XP patients with nodular melanoma is limited. In the absence of XP‐specific prognostic data, clinical outcomes are generally considered to depend on established melanoma prognostic factors, including tumor stage at diagnosis, Breslow thickness, ulceration, and timely treatment.

The occurrence of nodular melanoma in M3 is therefore consistent with the markedly increased melanoma susceptibility associated with XP, although the aggressive clinical course likely reflects both the intrinsic biological behavior of nodular melanoma and the underlying DNA repair deficiency [4, 26].

In 2011, at the age of 9 years, M3 was submitted to a chemotherapy protocol using CDDP, 5‐FU, and DCT to treat an extensive facial tumor. Since then, his clinical condition has worsened considerably, with an increase in tumor recurrences and reduced healing capacity. Consistent with previous reports, Zhang et al. [25] showed that XP‐C patients under CDDP chemotherapy—a cytotoxic agent that forms cross‐links and bulky DNA adducts that block replication and induce cell death—showed a progression similar to that observed in our XP‐V patients. This is unexpected for XP‐V, as these patients typically exhibit a milder phenotype compared with XP‐C. In the same study, the authors highlight that CDDP induced a severe adverse reaction that resulted in the death of both patients due to the damage originating from CDDP that is repaired by NER and TLS in normal cells, making the adverse effects fatal in XP patients [25].

In addition to CDDP, 5‐FU exerts its cytotoxic effect by inducing DNA damage indirectly. Specifically, its metabolite, FdUTP, is misincorporated into DNA in place of thymidine, resulting in base mismatches that trigger the base excision repair (BER) and mismatch repair (MMR) pathways in normal cells [27, 28]. However, should these repair mechanisms become overwhelmed or fail, resulting in the activation of TLS. In XP‐V patients, this pathway is compromised and the damage to noncancerous cells is greater, potentially exacerbating the patient′s clinical condition. These observations suggest that exposure to genotoxic therapies may have contributed to the different clinical courses observed between the twins. However, the specific contribution of CDDP cannot be determined, as detailed information regarding the chemotherapy regimen received by M6 was unavailable. Therefore, although M3 presented a more severe clinical course following CDDP‐based chemotherapy, additional factors, including cumulative UV exposure and other biological modifiers, may also have influenced disease progression. In contrast, DCT acts inhibiting mitosis by binding with high specificity to tubulin proteins and consequently prevents cell division, without acting at DNA damage level [27, 29].

An important limitation of this interpretation is the incomplete clinical information regarding M6′s oncological treatment. Although M3 received a documented CDDP‐based chemotherapy regimen, the specific agents and treatment schedules used for M6 were unavailable. Therefore, the contribution of individual chemotherapeutic agents to the differences observed in somatic mutational burden and clinical severity cannot be conclusively established.

According to Hummel′s classification, the MZ status of Patients M3 and M6 was confirmed with a > 99.9% confidence level using autosomal and Y‐STR genotyping and was important to help characterize and understand the clinical and genetic profile associated with the XP condition. This type of genotyping is widely used for human identification, for example, to indicate paternity [30].

The germinative mutation detected in these twins, an insertion *POLH* c.672_673insT was described for the first time in XP patients from the city of Montanhas (Rio Grande do Norte, Brazil), who have a milder phenotype [22]. This mutation is deleterious for gene function, as it culminates in a change in the amino acid reading frame from a leucine to a serine and protein truncation (p.Leu225Serfs ^∗^33). The p.Leu225Serfs ^∗^33 variant (rs772778835) was described by gnomAD as being of low frequency and present in a heterozygous state in both the Latin American and European populations.

Despite a similar *POLH* mutation of the genetic cluster of XP‐V patients identified in Montanhas, it was possible to observe that M3 presents a more severe clinical course [22]. M3, at age 28 years, underwent more than 30 surgeries to remove tumors and has severe complications, such as the absence of part of the right side of the face and reduced maxillary mobility. Castro et al. [22] also reported that only one of their XP‐V patients developed melanoma at age 72 years, whereas in the present study, M3 developed a nodular melanoma in his right eye at 20 years old. In contrast, Patient M6 presented a comparatively less clinical course, although metastatic disease was also observed. Together, these findings reinforce the absence of a straightforward genotype–phenotype correlation in XP‐V and suggest that clinical severity is influenced by additional genetic or environmental beyond the underlying *POLH* mutation [31].

An important consideration is that WGS was performed using DNA extracted from peripheral blood samples. Therefore, the somatic variants identified in this study represent systemic blood‐derived somatic alterations and may not fully capture the mutational landscape of the cutaneous tumors. Although these variants provide insights into the cumulative mutational processes occurring in the patients, tumor sequencing would be required to directly characterize the genomic alterations associated with individual carcinomas [32–34].

Our patients exhibit variations in the total number of unique somatic mutations: M3 carries 11,132, whereas M6 has 7781. Although both twins present mutational signatures compatible with chemotherapy, the documented chemotherapeutic protocol (CDDP + 5 − FU + DCT) is available only for M3. These mutational findings corroborate the studies by Hirsch et al. [35], Volkova et al. [36], and Boot et al. [37], they demonstrated in tumor cells that CDDP treatment can lead to an increased mutational burden associated with distinct signature patterns.

The predominance of C > T transitions in both patients, despite the higher absolute variant count in M3 (3208) than in M6 (2171), reinforces the similarity in their mutational profiles. The distribution of these substitutions, which ranged from 31% to 34.1% in CpG sites and from 52.4% to 56% in YpY regions. For all other contexts, the distribution remained consistent, ranging from 12.9% to 13.4% in both patients. The typical Ti/Tv ratio for human genomes ranges approximately between 2.1 and 2.7 [38] however, our patients presented ratios of 1.02 for M3 and 0.96 for M6. This deviation suggests specific mutagenic processes, as Alexandrov et al. [39] point out that different mutational signatures generated by DNA damage can significantly alter the proportion of transitions and transversions.

Furthermore, the proximity of the Ti/Tv ratios between the XP‐V twins suggests the occurrence of similar mutagenic processes, supported by the high correlation of their SBS spectra (cosine similarity *r* > 0.9). Despite the WGS being performed on blood samples, our findings corroborate the work of Degasperi et al. [40], who observed in tumor samples the presence of similar signatures even when the mutational burden differs between individuals, as seen in our patients.

The number of deletions presented by the patients diverges, with M3 exhibiting 4039 and M6 showing 3541. These results corroborate the findings of Wang et al. [41], who point out that CDDP tends to induce the formation of intra‐ and interstrand DNA crosslinks, which block the replication fork. According to the same authors, such damage can result in DSBs that are subsequently repaired by NHEJ; however, this repair process is prone to generating small deletions during DNA ligation.

Patients M3 and M6 exhibit similar signature contribution profiles; however, there is a divergence in the percentages of these contributions. M6 shows a 60.5% contribution from SBS3 and 39.5% from SBS25, whereas M3 presents 41.6% and 27.7%, respectively. Additionally, there are exclusive signatures in M3, namely SBS6 (14.1%) and SBS37 (16.6%). SBS3 is a robust marker of deficiency in homologous recombination DNA repair (HRD) [39, 42]; its presence indicates genomic instability in these patients, likely arising from compensatory repair pathways. Furthermore, Alexandrov et al. [39] and Otlu et al. [43] discuss that SBS25 is associated with exogenous processes such as chemotherapy exposure. Although previously observed in Hodgkin lymphoma–derived cell lines, its presence corroborates the patients′ chemotherapy history. Nevertheless, Hu et al. [44] discuss that the complete etiology of SBS25 remains unknown, potentially being associated with rare mutational processes or specific environmental exposures. Finally, SBS6 and SBS37 present only in M3, are associated with MMR deficiency and mutational processes that are not yet fully elucidated [39]. This exclusive finding in M3 may indicate an even greater degree of genomic instability.

The twins, M3 and M6, share only one COSMIC Indel signature, ID2, though with distinct contributions: 14.6% for M3 and 30.2% for M6. Regarding the other signatures, M3 exhibits a 44.3% contribution from ID8 and 40.8% from ID14. In contrast, M6 presents a 25% contribution from ID1 and 44.8% from ID12. ID1 and ID2 are common signatures in several cancer types and are associated with polymerase slippage during DNA replication at homopolymer tracts [39, 45]. Furthermore, Thatikonda et al. [45] suggest that ID8 may result from DNA damage induced during the repair of DSBs via NHEJ, as this mechanism can generate small insertions and deletions during the DNA ligation process [46]. In the context of our study, the presence of this signature may also be associated with M3′s clinical history of CDDP treatment. Since CDDP induces interstrand cross‐links that often lead to the formation of DSBs, the subsequent recruitment of the NHEJ pathway for repair could further contribute to the observed ID8 pattern. Finally, the etiologies of ID12 and ID14 remain poorly defined [15, 47].

Results obtained using the pLogo method [16] revealed statistically significant sequence motifs for C > A, C > T, T > A, and T > C substitutions (*p* < 0.05). Similar motifs were observed for the occurrence of C > A, with both being more frequent in regions A‐rich. Furthermore, for the occurrence of C > T, M3 and M6 show overrepresentation of G the +1 position. In turn, for the T > C and T > G variants, similar motifs were also observed for M3 and M6, this time, in T‐rich regions. These findings also reinforce the genomic similarities between the patients, suggesting that both share the same underlying mutational processes. This similarity can be attributed also to endogenous DNA damage, which are by‐products of cell metabolism, during the lifetime of these patients. The absence of Pol *η* may have contributed to at least part of these mutations. Thus the conservation of sequence motifs across M3 and M6 indicates that Pol *η* deficiency, coupled with their chemotherapeutic history, operates through similar mechanisms, despite the variation in total mutational burden observed between the individuals. Degasperi et al. [40] and Alexandrov et al. [39], demonstrated that specific mutational processes produce distinct substitution patterns that are highly dependent on the sequence context surrounding the altered nucleotide.

To mimic the patients′ condition and understand the impact of CDDP on XP‐V individuals, TLS deficient cells were utilized. Our data demonstrated that XP‐V cells, particularly after prolonged exposure (72 h) or with increasing doses of CDDP (up to 10 *μ*M), show a dramatic reduction in viability, with survival dropping to approximately 4% at a concentration as low as 1 *μ*M. These findings were especially evident when compared with negative controls or cells complemented with functional Pol *η*. Albertella et al. [48] and Bassett et al. [49] also demonstrated that TLS‐deficient cells are approximately three times more sensitive to CDDP. The authors also discussed that when these cells are complemented with a functional *POLH* gene, sensitivity is reduced to normal levels, consistent with our observations in XP‐V comp cells.

Cell cycle progression was not affected 24 h after CDDP exposure; after 72 h, cell recovery is evident in the XP‐V comp line; however, the XP‐V line remains stalled at the G1/S transition, failing to re‐enter the cell cycle. These data corroborate the studies by Anand et al. [50] and Lord and Ashworth [51], as the absence of Pol *η* can lead to replication stress and activation of cell cycle checkpoints, resulting in the observed G1/S arrest. Panaretakis [52] and Elmorsy et al. [53] suggest that when DNA damage remains unrepaired, DNA fragmentation increases, leading the cells to enter apoptosis.

Recent clinical reports have raised important concerns regarding the safety of CDDP‐based chemotherapy in patients with XP. Sumiyoshi et al. [54] described catastrophic and ultimately fatal adverse reactions following standard cisplatin‐containing regimens in XP patients, attributing this increased susceptibility to defective NER and TLS pathways, which normally protect healthy tissues from platinum‐induced DNA damage. More recently, Gilbar and Pokharel [55] reported severe cisplatin‐induced acute kidney injury in a patient with XP receiving adjuvant chemoradiotherapy for cutaneous squamous cell carcinoma, further supporting the exceptional vulnerability of XP patients to platinum‐associated toxicity. These clinical observations are consistent with experimental studies demonstrating that *XPA* and *POLH* deficient cells exhibit increased sensitivity to CDDP, highlighting the critical role of DNA repair pathways in limiting treatment‐induced cytotoxicity. In agreement with these findings, our cell viability and cell cycle assays demonstrated increased sensitivity of XP‐V cells to CDDP, providing mechanistic evidence that supports the growing clinical concern regarding the use of platinum‐based chemotherapy in this population. Although our study cannot establish a direct causal relationship between CDDP exposure and the more severe clinical course observed in M3, our findings, together with the available clinical evidence, support careful risk‐benefit assessment when considering platinum‐based chemotherapy in patients with XP. Whenever clinically appropriate and effective alternatives are available, non‐DNA–damaging therapeutic strategies, including immune checkpoint inhibitors, should be preferred.

## 5. Conclusions

Despite sharing the same homozygous *POLH* pathogenic variant and a highly similar somatic mutational landscape, the MZ twins exhibited markedly different clinical courses, highlighting that disease severity in XP‐V is likely influenced by genetic and nongenetic modifiers beyond the causal variant. Our functional assays demonstrated increased cellular sensitivity to CDDP, providing mechanistic evidence that impaired TLS enhances vulnerability to platinum‐induced DNA damage, consistent with previous clinical reports of severe cisplatin‐associated toxicity in XP patients. Taken together with the growing clinical evidence, our findings provide additional mechanistic support for avoiding platinum‐based chemotherapy whenever effective alternative treatments, including immune checkpoint inhibitors or other non‐DNA–damaging therapies, are available. Finally, our study underscores the importance of early molecular diagnosis to guide personalized clinical management, optimize therapeutic decision‐making, and improve long‐term outcomes in patients with XP.

## Author Contributions

J.P.D.S.J., L.G.S., C.F.M.M., and J.A.S. designed the study. J.P.D.S.J. and V.M. performed the data collection and processing of the biological samples. J.A.S., L.G.S., and C.F.M.M. assisted in conducting the study and processing the data. V.Q.B. performed the interpretation of the genotyping. J.P.D.S.J., M.C.F.C.S., C.C., W.J.S.J., and T.L.C. performed whole genome sequencing and bioinformatics analyses. M.C.F.C.S. was responsible for identifying the mutational signatures. J.P.D.S.J., M.C.F.C.S., L.G.S., C.F.M.M. and J.A.S. wrote the manuscript. All authors have reviewed the manuscript. J.P.D.S.J. and M.C.F.C.S. contributed equally to this work. C.F.M.M. and J.A.S equally managed the project, study assays, manuscript queries and should be regarded as senior co‐corresponding authors. J.P.D.S.J. and M.C.F.C.S. are the first coauthors.

## Funding

This work was supported by the FAPESP (Sao Paulo Research Foundation, São Paulo, Brazil) (Grants #2019/19435‐3, #2013/08028‐1, #2023/09586‐0), Conselho Nacional de Desenvolvimento Científico e Tecnológico (CNPq, Brasília, Federal District, Brazil) (Grants #308868/2018‐8, #444181/2023‐7, #403844/2021‐5, #300025/2025‐4), Coordenação de Aperfeiçoamento de Pessoal de Nível Superior (CAPES) (Brasília, Federal District, Brazil, Financial Code 001) (Grant No. 88887.637286/2021‐00) (Programa Demanda Social), and Pernambuco State Foundation for Science and Technology Support (FACEPE) (IBPG‐0115‐2.00/24).

## Disclosure

All authors approved the final version of this document.

## Ethics Statement

This study was approved by the Research Ethics Committee of the Federal University of Pernambuco (CAAE: 51770621.0.0000.5208), and by the ethics committees from Institute of Biomedical Sciences (CAAE: 48347515.3.0000.5467), University of São Paulo.

## Conflicts of Interest

The authors declare no conflicts of interest.

## Supporting information


**Supporting Information** Additional supporting information can be found online in the Supporting Information section. Figure S1: Geographic distribution of cities associated with the XP family history in Pernambuco, Brazil. Table S1: Summary of somatic single nucleotide variants (SNVs) identified in Patients M3 and M6 before and after exclusive variant filtering. Table S2: Summary of somatic insertions and deletions (indels) identified in Patients M3 and M6 before and after exclusive variant filtering. Table S3: Frequency and distribution of C > T transitions by mutational context. Table S4: Dominant trinucleotide mutational contexts in M3 and M6.

## Data Availability

The data that support the findings of this study are openly available in Sequence Read Archive (SRA) at https://www.ncbi.nlm.nih.gov/sra/PRJNA1471031 (Reference Number PRJNA1471031).
