# Supplementary material for: Xeroderma Pigmentosum Monozygotic Twins: Same Mutation Divergent Clinical Outcomes
Source: Hum Mutat. 2026 Oct 7;2026:7247751. doi: 10.1155/humu/7247751 (PMC13643933; doi:10.1155/humu/7247751)
Supplement: Supplementary file 1 [file HUMU-2026-7247751-s001.pdf]

Figure S1 - Geographic distribution of cities associated with the XP family history in Pernambuco, Brazil.

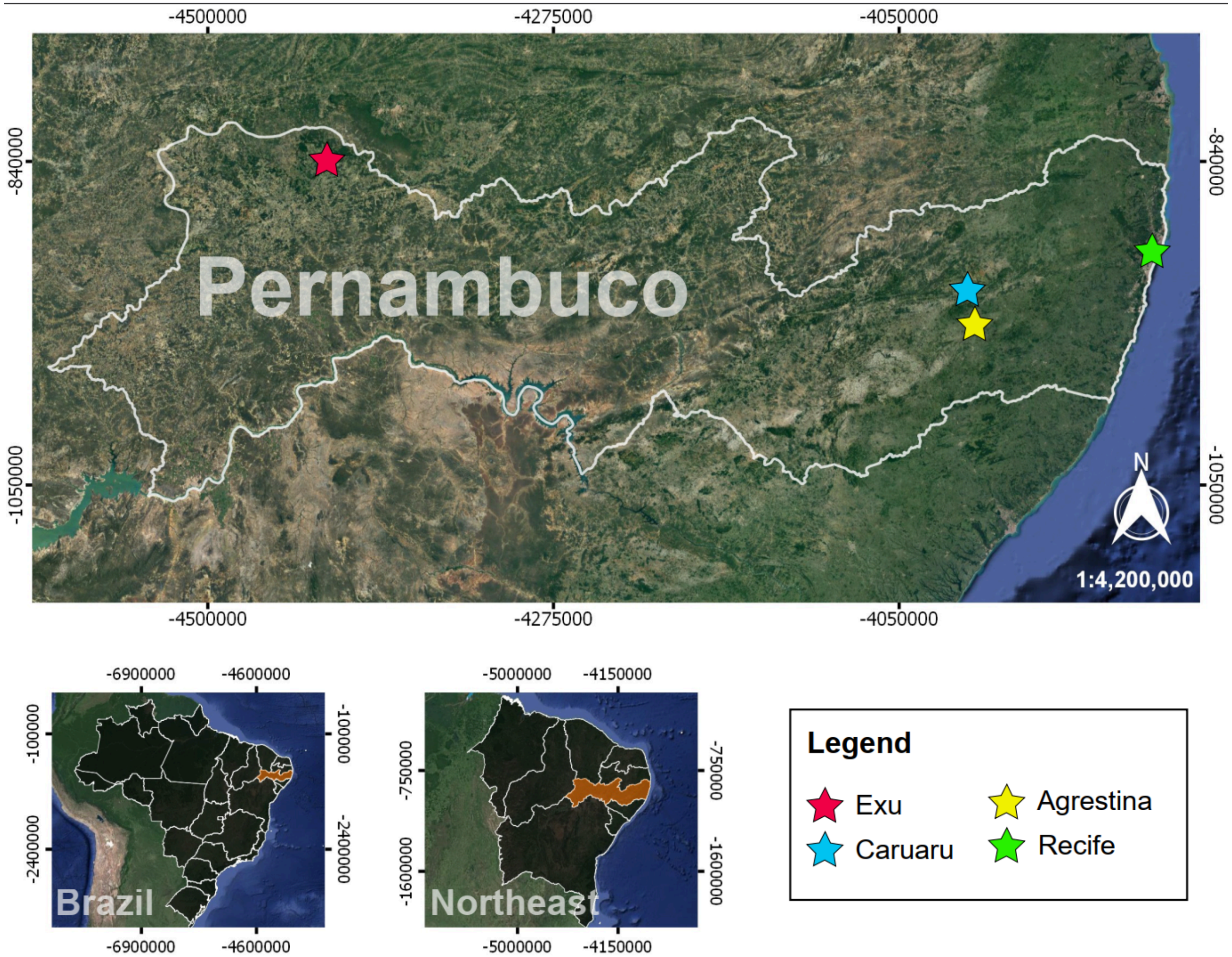

Legend: The patients' family migrated from Exu (birthplace of the great-aunt with a clinical history suggestive of XP) to Agrestina, where the family currently resides. The patients were born in Caruaru. Regional climatological UV index data (TEMIS, 2025) indicate persistently very high to extreme UV index values radiation throughout Pernambuco and Northeastern Brazil (UV index approximately 8 in winter and 12-14 during the remaining seasons). These data provide regional environmental context regarding ambient UV radiation levels but do not represent individual cumulative lifetime UV exposure.

Table S1 - Summary of somatic single nucleotide variants (SNVs) identified in patients M3 and M6 before and after exclusive variant filtering.

SNVs before filtering for exclusive variants - Total variants

Sample	Total Variants	SNVs Count	Ti/Tv Ratio	C>A	C>G	C>T	T>A	T>C	T>G	exonic	splicing	intronic	UTR3	UTR5	intergenic	upstream	downstream	NcRNA exonic	NcRNA intronic
M3	47.047	47.047	1.46	5.266	4.629	15.575	5.125	12.380	4.072	169	2	8.503	335	62	34.182	259	236	213	3.072
M6	47.710	47.710	1.49	5.310	4.737	15.760	4.962	12.770	4.171	193	2	8.585	318	61	34.716	272	273	205	3.075

SNVs after filtering for exclusive variants - Exclusive variants

Sample	Total Variants	SNVs Count	Ti/Tv Ratio	C>A	C>G	C>T	T>A	T>C	T>G	exonic	splicing	intronic	UTR3	UTR5	intergenic	upstream	downstream	NcRNA exonic	NcRNA intronic
M3	11.132	11.132	1.02	1.340	1.260	3.208	1.850	2.404	1.070	22	0	1.550	77	8	8.515	58	54	66	776
M6	7.781	7.781	0.96	1.004	909	2.171	1.294	1.637	766	33	0	1.060	47	6	6.030	52	42	40	470

Legend: Whole-genome sequencing (WGS) data were analyzed to compare variant profiles between the twins. The table includes the total number of SNVs, transition/transversion (Ti/Tv) ratios, possible nucleotide substitution classes, and genomic distribution of variants.

Table S2 - Summary of somatic insertions and deletions (indels) identified in patients M3 and M6 before and after exclusive variant filtering.

INDELs before filtering for exclusive variants - Total variants

Sample	Total InDels	Insertions	Deletions	exonic	intronic	intergenic	splicing	UTR3	UTR5	Frameshift delrtion	Frameshift Insertion
M3	13.217	6.784	6.433	13	4.582	7.395	0	123	6	7	3
M6	15.954	8.902	7.052	11	5.730	8.705	0	133	10	7	3

INDELs after filtering for exclusive variants - Exclusive variants

Sample	Total InDels	Insertions	Deletions	exonic	intronic	intergenic	splicing	UTR3	UTR5	Frameshift Deletion	Frameshift Insertion
M3	7.442	3.403	4.039	8	2.854	3.928	0	79	3	7	0
M6	7.611	4.070	3.541	8	3.007	3.891	0	60	4	7	0

Legend: WGS data were analyzed to compare indel profiles between the twins. The table includes the total number of insertions and deletions, their individual counts, and the genomic distribution of variants.

Table S3. Frequency and Distribution of C>T Transitions by Mutational Context.

C>T Transition Context

Relative distribution within the C>T mutation class (M3 vs M6)

Context	M3 (N)	M6 (N)	M3 (%)	M6 (%)
**Total C>T**	**3208**	**2171**	**100.00**	**100.00**
C>T in CpG	*1096*	*673*	*34.16*	*31.00*
C>T in YpY	*1682*	*1217*	*52.43*	*56.06*
Other C>T	*430*	*281*	*13.40*	*12.94*

Legend: Absolute frequencies and relative contributions (%) of C>T transitions in patients M3 and M6, categorized according to CpG sites, pyrimidine-pyrimidine (YpY) regions, and other genomic contexts.

Table S4. Dominant Trinucleotide Mutational Contexts in M3 and M6.

Dominant trinucleotide mutational contexts				
**Triplet**	**M3 (%)**	**M6 (%)**	**M3 (N)**	**M6 (N)**
A(C>T)G	3.14	3.02	349	235
A(C>T)A	2.58	2.29	287	178
G(C>T)G	2.43	2.39	271	186
A(T>C)A	2.42	1.88	269	146
C(C>T)G	2.25	1.66	251	129
T(T>C)T	2.16	1.91	240	149
T(T>A)A	2.09	1.54	233	120
T(C>T)G	2.02	1.58	225	123
A(T>C)G	2.01	1.98	224	154
T(C>T)T	1.84	2.34	205	182
C(C>T)T	1.75	2.18	195	170

Legend: Relative contribution (%) and absolute number (N) of dominant trinucleotide mutational contexts identified in patients M3 and M6. The table includes the trinucleotide contexts with the highest relative contributions across the 96 single-base substitution (SBS) categories.
